# Vimentin-mediated buffering of internal integrin β1 pool increases survival of cells from anoikis

**DOI:** 10.1186/s12915-024-01942-w

**Published:** 2024-06-24

**Authors:** Jiyoung Jang, Hyun Jung Park, Wonyoung Seong, Jiyoon Kim, Chungho Kim

**Affiliations:** 1 Department of Life Sciences, Korea University, Seoul, 02841 Republic of Korea; 2 Samsung Genome Institute, Samsung Medical Center, Seoul, 06351 Republic of Korea; 3 Donnelly Centre, University of Toronto, ON Toronto, M5S 3E1 Canada

**Keywords:** Integrin, Vimentin, Anoikis, Metastasis, Epithelial-to-mesenchymal transition

## Abstract

**Background:**

The intermediate filament protein vimentin is widely recognized as a molecular marker of epithelial-to-mesenchymal transition. Although vimentin expression is strongly associated with cancer metastatic potential, the exact role of vimentin in cancer metastasis and the underlying mechanism of its pro-metastatic functions remain unclear.

**Results:**

This study revealed that vimentin can enhance integrin β1 surface expression and induce integrin-dependent clustering of cells, shielding them against anoikis cell death. The increased integrin β1 surface expression in suspended cells was caused by vimentin-mediated protection of the internal integrin β1 pool against lysosomal degradation. Additionally, cell detachment was found to induce vimentin Ser38 phosphorylation, allowing the translocation of internal integrin β1 to the plasma membrane. Furthermore, the use of an inhibitor of p21-activated kinase PAK1, one of the kinases responsible for vimentin Ser38 phosphorylation, significantly reduced cancer metastasis in animal models.

**Conclusions:**

These findings suggest that vimentin can act as an integrin buffer, storing internalized integrin β1 and releasing it when needed. Overall, this study provides insights regarding the strong correlation between vimentin expression and cancer metastasis and a basis for blocking metastasis using this novel therapeutic mechanism.

**Supplementary Information:**

The online version contains supplementary material available at 10.1186/s12915-024-01942-w.

## Background

Vimentin is a type III intermediate filament protein that is widely expressed in mesenchymal cells [1]. It consists of two coiled-coil domains involved in parallel dimer formation, flanked by two nonstructural head and tail domains [2]. Two vimentin dimers associate to form an antiparallel tetramer that can further assemble both laterally and longitudinally to form a filamentous structure [3, 4]. The resulting vimentin filaments regulate cell shape, motility, and adhesion [5].

Vimentin is a well-known marker of the epithelial-to-mesenchymal transition (EMT), which is the initial critical phase of metastasis [1]. Several studies have demonstrated that the EMT-promoting function of vimentin is associated with poor clinical outcomes in various cancers, including leukemia [6], lung [7], neuroendocrine [8], gastric [9], colorectal [10, 11], cervical [12], and breast [13] cancer. Evidence supports that the expression of vimentin leads to the conversion of benign tumor cells into metastatic cells with an increased invasive phenotype [14, 15], whereas vimentin deficiency results in various defects in EMT-promoting cellular architectures, including impaired microtubule polarization, failure to form actin stress fibers, and defective focal adhesion maturation, which lead to reduced mechanical strength [16]. This strong association between vimentin and the metastatic potential of cancer makes it a promising drug target for cancer therapy [1, 17].

Although a large number of studies exist on the involvement of vimentin in cancer metastasis, studies focusing on the underlying mechanisms of its pro-metastatic function are limited. Nonetheless, it appears that the EMT-related function of vimentin may depend on its promotion of cell–matrix interactions over cell–cell interactions. For example, we previously demonstrated that vimentin filaments can increase their adhesiveness toward the extracellular matrix (ECM) and favor the EMT process by binding to integrin β1 and β3 cytoplasmic tails, inducing their clustering [18, 19]. Vimentin filaments are also known to act as adaptors that recruit focal adhesion proteins to integrin-ECM contacts for enhanced cell migration [20]. In addition, tyrosine phosphorylation by growth factor signaling changes vimentin filament dynamics, activating the Vav2-Rac1 signaling pathway and leading to lamellipodia formation and cell migration [21].

The pro-metastatic function of vimentin may not be restricted to the regulation of EMT. A recent study suggested that vimentin can delay apoptosis progression by modulating the transcription of the pro-survival factor NFκB [22]. Moreover, when phosphorylated by Plk1, vimentin activates cMET and enhances the survival of mesenchymal non-small cell lung cancer cells [23]. Consistently, knockdown of vimentin expression significantly increases lymphocyte apoptosis in the context of sepsis, whereas vimentin overexpression results in the opposite outcome [24]. Since cancer cells need to survive even in the absence of proper cell adhesion in the bloodstream during their journey to distant metastatic sites, we hypothesized that the survival benefits of vimentin may reduce programmed cell death of cancer cells, or anoikis, caused by the absence of adhesion [25, 26]. Therefore, we investigated the possible protective roles of vimentin against anoikis cell death and its underlying mechanism, which can explain further, in a different point of view rather than that related to its EMT promoting roles, why vimentin expression is closely related to successful metastasis.

In the present study, we demonstrated that the presence of vimentin in suspended cells can enhance the surface expression of integrin β1 and the integrin-mediated clustering of cells, leading to improved cell survival in suspension. We have also shown that vimentin can trap internal integrin β1, protecting it from lysosomal degradation. Additionally, vimentin phosphorylation caused by cell detachment results in integrin’s translocation to the cell surface. Based on these results, we propose that the ability of vimentin to buffer internal integrin promotes metastasis. The mechanistic insights provided by this study constitute the basis for a potential therapeutic approach used for the prevention of cancer metastasis.

## Results

### Vimentin reduces anoikis cell death

**Fig. 1:**
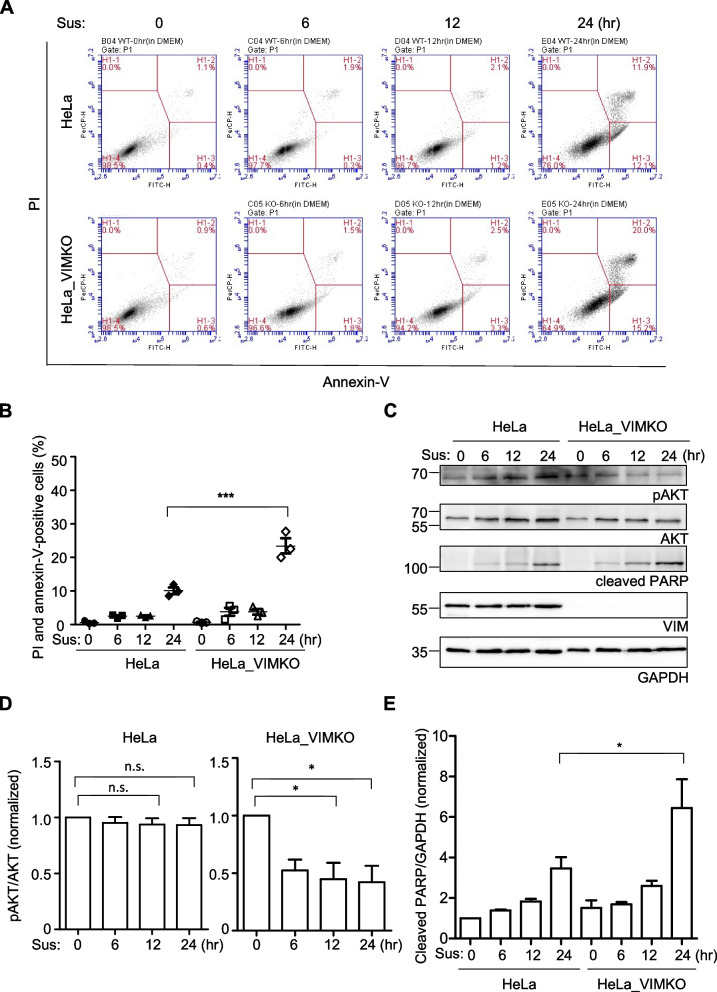
The effects of vimentin on anoikis cell death. **A** Wild-type HeLa cells and HeLa_VIMKO cells were suspended for the indicated time, stained with annexin-V and propidium iodide (PI), and analyzed under flow cytometry. **B** Percentages of both PI and annexin-V-positive cells in (**A**) are shown as a graph. ***, p < 0.0001 (one-way ANOVA followed by Tukey multiple-comparison test, *n* = 3). **C** HeLa and HeLa_VIMKO cells suspended for the indicated time were analyzed for their phospho-AKT and cleaved PARP levels by western blot. AKT and GAPDH were also analyzed as loading controls. **D** The ratio of phospho-AKT to AKT levels at each time point was normalized against that in a 0-h suspension for wild-type and HeLa_VIMKO cells, respectively. ∗ , *p* < 0.05, n.s., not significant (one-way ANOVA followed by Tukey multiple-comparison test, *n* = 3). **E** The normalized ratio of cleaved PARP to GAPDH at each time point is shown. ∗ , *p* < 0.05 (one-way ANOVA followed by Tukey multiple-comparison test, *n* = 3)

To investigate the pro-metastatic protective role of vimentin against anoikis, we generated vimentin knockout HeLa cells (HeLa_VIMKO) via CRISPR-Cas9 technology (Additional file 1: Supplementary Fig. 1A and 1B). The targeted decrease in vimentin expression was validated through RNA sequencing analysis (Additional file 1: Supplementary Fig. 1C and 1D). Subsequently, we compared the survival of HeLa_VIMKO cells to that of parental cells using an in vitro model employing poly-2-hydroxyethyl methacrylate (poly-HEMA) to create a non-cell-binding hydrogel on dishes [27]. Suspensions of HeLa and HeLa_VIMKO cells were incubated for 0–24 h on the poly-HEMA-coated surface. Their binding to annexin V, a marker of apoptosis, and incorporation of propidium iodide (PI), a marker of cell death [28], were measured using flow cytometry (Fig. 1A). As expected, suspension time was positively associated with the number of apoptotic wild-type HeLa cells, which were positive for both PI and annexin V (Fig. 1A). The absence of vimentin expression further increased apoptotic cell death (Fig. 1B), suggesting a beneficial role of vimentin under anoikis conditions. We next compared the representative survival signal, phosphorylated Akt [29], and the apoptotic signal, cleaved poly(ADP-ribose) polymerase (PARP) produced by caspase [30] (Fig. 1C). While AKT phosphorylation relative to AKT expression in wild-type HeLa cells remained unchanged for up to 24 h in suspension (Fig. 1D, left), the same ratio in HeLa_VIMKO cells dropped significantly as early as 6 h after suspension (Fig. 1D, right). Apoptotic signaling, represented by cleaved PARP, was also consistently increased in HeLa_VMKO cells compared to that in wild-type cells (Fig. 1E). Taken together, these results suggest that vimentin plays a protective role against anoikis cell death, thereby potentially facilitating cancer cell metastasis.

### Vimentin expression positively correlates with the expression of ECMs but not integrins in circulating tumor cells

**Fig. 2:**
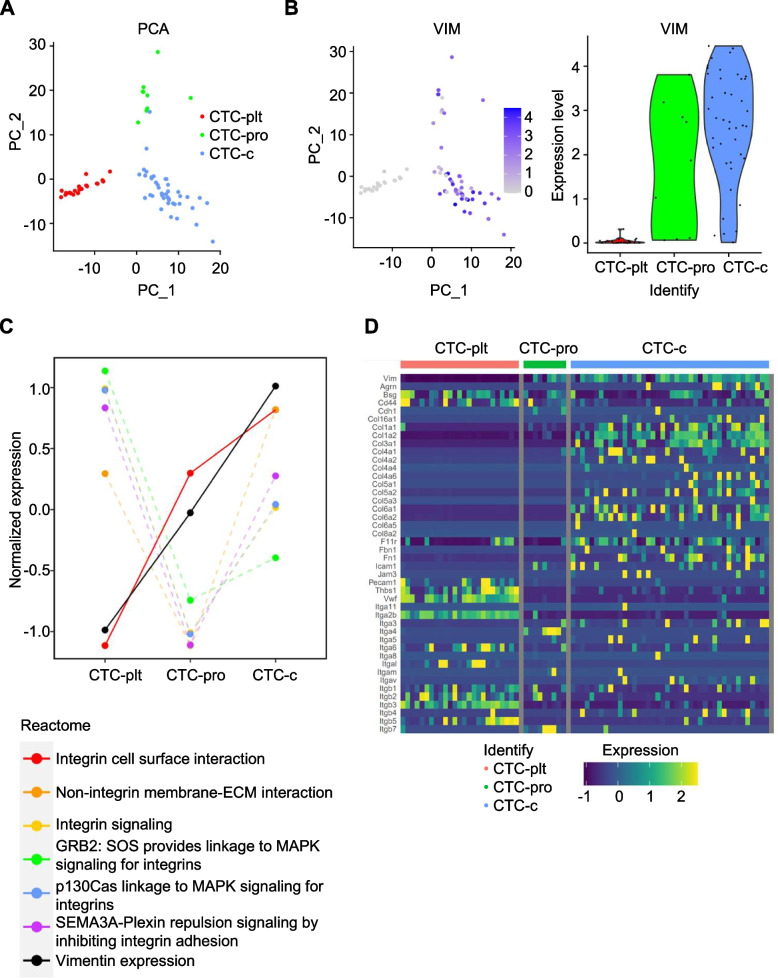
Correlation of vimentin with extracellular matrices in circulating tumor. **A** Principal component analysis (PCA) was done on the single-cell RNA sequencing results of the pancreatic circulating tumors (CTCs) from a publicly available database. The red, green, and blue dots represent CTCs originally described as platelet-adhered CTCs (CTC-plt), proliferative CTCs (CTC-pro), and classical CTCs (CTC-c), respectively. **B** Relative expression of Vim in CTCs visualized in PCA space (left) and as violin plots for CTC types (right). **C** Reactome analysis. The pathway-level gene expressions (integrin-related) and Vim expression (black) for CTC types. **D** Heatmap of highly variable genes in the ‘Integrin cell surface interactions” pathway

To get a clue on this vimentin-dependent survival benefit in suspended cells, we first analyzed a publicly-available single cell RNA sequencing experiment conducted on pancreatic circulating tumor cells (CTC) [31]. As in the study, our independent principal component analysis (PCA) identified three different groups of CTCs: platelet-adhered CTCs (CTC-plt), proliferative CTCs (CTC-pro), and classical CTCs (CTC-c) (Fig. 2A). In these groups, vimentin expression was most frequently, moderately, and rarely observed in CTC-c, CTC-pro, and CTC-plt, respectively (Fig. 2B). We conducted a pathway analysis focused on integrin-related pathways based on our previous finding on vimentin-mediated integrin clustering [18, 19], which may help sustain integrin-dependent survival signaling. Interestingly, our analysis using the Reactome database [32] revealed that the vimentin expression pattern was positively correlated with the pathway termed “integrin cell surface interactions” (Fig. 2C). Subsequent analysis of individual genes in the pathway revealed a sharp positive correlation between the expression of vimentin and ECM components such as collagen and fibronectin in individual CTCs within these groups (Fig. 2D). However, no clear relationship was observed in the expression of their receptor integrins (Fig. 2D). An exception was observed in platelet integrins αIIb and β3, although their expression could be attributed to platelets attached to rather than from CTC-plt itself. Although the data from the CTCs from different cancer model may not reflect general behavior of metastatic cancer cells, the high level of ECM proteins in the vimentin-expressing CTC-c led us to hypothesize that these proteins may act as a type of glue that allows the CTCs to adhere to each other, thereby increasing their survival in non-adherent environments.

### The survival benefit of vimentin in suspended cells depends on integrin

**Fig. 3:**
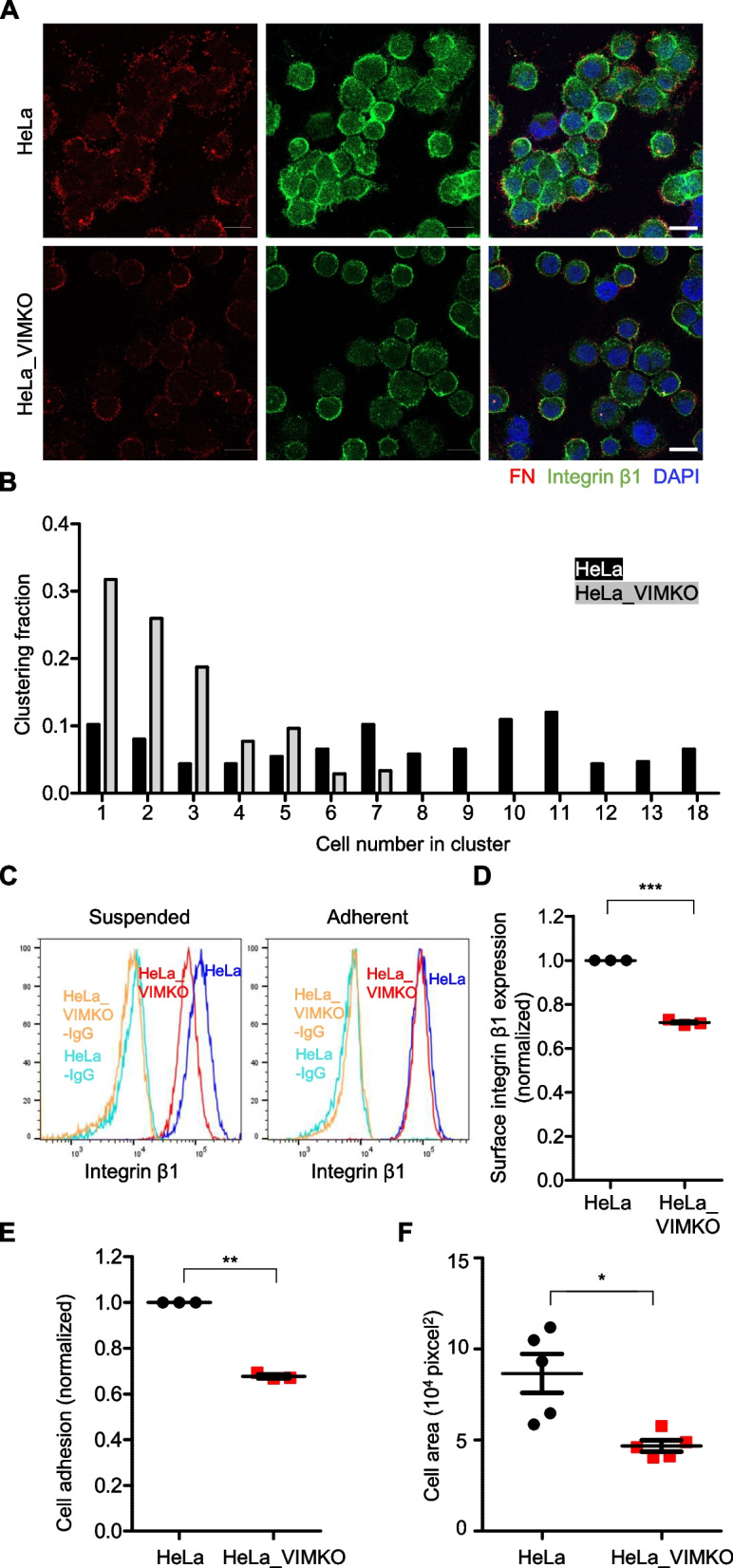
Vimentin-dependent clustering of suspended cells. **A** HeLa and HeLa_VIMKO cells were suspended for 24 h, fixed, and then stained with antibodies against fibronectin (red), integrin β1 (green), and DAPI for the nucleus (blue), followed by analysis with laser confocal scanning microscopy. Representative images are shown. Scale bar, 20 μm. **B** The number of cells in each cell cluster was counted, and the distribution of clusters based on the number of cells is presented as a bar graph. **C** Surface integrin β1 levels in HeLa or HeLa_VIMKO cells were analyzed after suspension for 24 h (left) or right after detachment (right side), and shown as histograms. Histograms of cells stained with mouse IgG are also shown as negative controls. **D** The mean fluorescence intensities of integrin β1 staining in each cell that was suspended for 24 h were normalized against those of wild-type HeLa cells and presented as a one-dimensional scatter plot. ***, *p* < 0.0001 (paired T test, *n* = 3). **E** The relative degrees of adhesion to the fibronectin-coated surface (normalized against that to the poly-L-lysine-coated surface) of wild-type HeLa and HeLa_VIMKO cells suspended for 24 h are shown. **, *p* < 0.01 (paired T test, *n* = 3). **F** Wild-type HeLa and HeLa_VIMKO cells were suspended for 24 h, and their degrees of spreading (mean spreading area of cells in 5 different fields) on a fibronectin-coated surface are shown. *, *p* < 0.05 (paired T test)

To test the above hypothesis, fibronectin (Fig. 2D, Fn1), an ECM enriched in CTC-c, was stained in wild-type and vimentin-knockout HeLa cells suspended for 24 h on the poly-HEMA-coated surface. In contrast to HeLa_VIMKO cells, most wild-type cells readily formed clusters (Fig. 3A). Specifically, approximately 90% of the wild-type cells in suspension were found in clusters comprising more than two cells, whereas the ability of HeLa_VIMKO cells to form such clusters was reduced to less than 70% (Fig. 3B). Even when HeLa_VIMKO cells formed clusters, the number of cells in these clusters was significantly lower than that of wild-type cells (Fig. 3B). As expected in the RNA sequencing data, we observed that the fibronectin staining in wild-type HeLa was much more intense than that in HeLa_VIMKO cells (Fig. 3A). In addition, the well-known receptor for fibronectin [33] integrin β1 was detected at the site of cell–cell contact within the clusters (Fig. 3A), suggesting that integrin-fibronectin interaction may be involved in the clustering of suspended cells.

However, despite the lack of correlation between vimentin and integrin gene expression in the RNA-sequencing data (Fig. 2D), we noticed that the integrin β1 staining in wild-type HeLa was much more intense than that in HeLa_VIMKO cells (Fig. 3A). Flow cytometry-based analysis confirmed the reduced surface expression of integrin β1 in HeLa_VIMKO compared to that in wild-type cells after 24 h of suspension (Fig. 3C, left, and D). Interestingly, no such difference was observed in cells in the adherent state (Fig. 3C, right), suggesting that the increasing effect of vimentin on integrin β1 surface expression was initiated after cell detachment. Cell adhesion (Fig. 3E) and spreading (Fig. 3F) measurements on the fibronectin-coated surface, the well-known consequences of integrin signaling, confirmed the increased levels of surface integrin β1 in wild-type HeLa compared to HeLa_VIMKO cells.

**Fig. 4:**
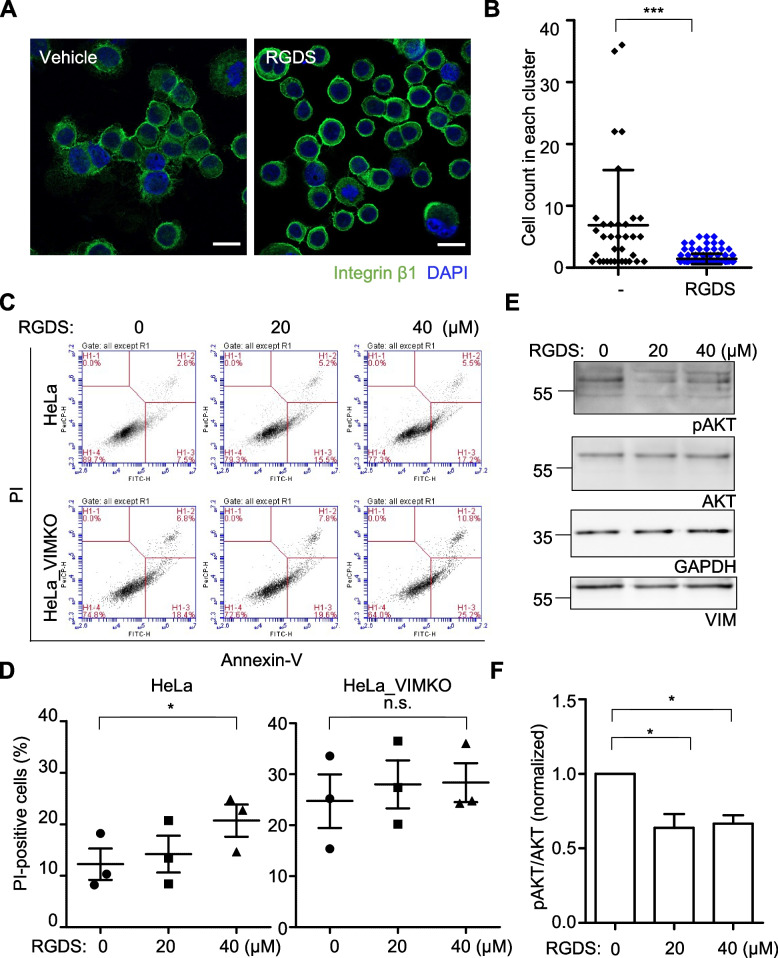
Integrin-dependent clustering and survival of suspended cells. **A** HeLa cells were suspended for 24 h in the presence of 40 μM RGDS or vehicle (PBS) and analyzed as in Fig. 3A. Scale bar, 20 μm. **B** The numbers of cells in cell clusters in each condition are shown as a one-dimensional scatter plot. ***, *p* < 0.0001 (paired T test). **C** HeLa and HeLa_VIMKO cells suspended for 24 h in the presence of the indicated amount of RGDS were analyzed as in Fig. 1A. **D** Percentages of PI-positive cells in (**C**) are shown as a graph. *, *p* < 0.05, n.s., not significant (paired T-test, *n* = 3). **E**, **F** In HeLa cells suspended for 24 h in the presence of the indicated amount of RGDS, the degrees of AKT phosphorylation normalized against AKT level in each condition were measured and analyzed as Fig. 1C and D. ∗ , *p* < 0.05 (one-way ANOVA followed by Tukey multiple-comparison test, *n* = 3)

The ligand-mimetic Arg-Gly-Asp-Ser (RGDS) peptide, which competitively inhibits the integrin β1-fibronectin interaction, was utilized to investigate the role of integrin surface expression in cell clustering and the survival of suspended cells [34, 35]. The addition of the RGDS peptide to wild-type HeLa cells significantly reduced cell clustering (Fig. 4A) and the number of cells per cluster during their 24-h suspension (Fig. 4B), confirming that integrin is involved in cluster formation. Moreover, the presence of RGDS peptide during 24-h suspension conditions significantly increased apoptosis in wild-type HeLa (Fig. 4C and D), but not in HeLa_VIMKO cells. In addition, the survival signal measured by Akt phosphorylation was also blocked by the RGDS peptide in suspended wild-type HeLa cells (Fig. 4E and F). Overall, our results suggest that the presence of vimentin in suspended cells can cause enhanced integrin β1 surface expression, leading to enhanced cell clustering via fibronectin and triggering survival signaling in the suspended cells.

### Vimentin prevents degradation of internal integrin β1

**Fig. 5:**
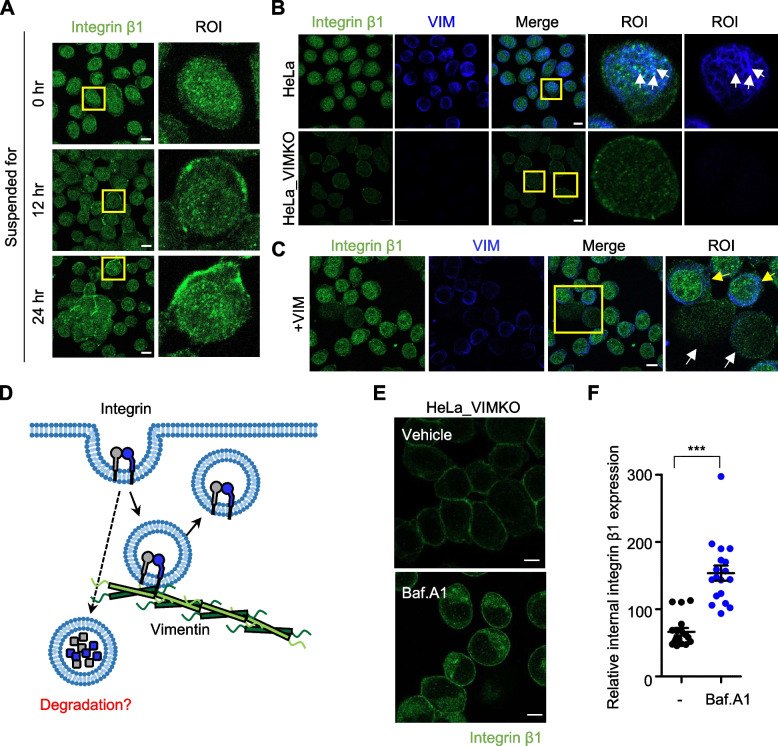
Protection of internal integrin β1 by vimentin. **A** HeLa cells suspended for 0, 12, or 24 h were stained with anti-integrin β1 (green). The yellow boxes represent regions of interest (ROI) that have been digitally magnified on the right. Scale bar, 10 μm. **B** HeLa cells and HeLa_VIMKO cells were stained for integrin β1 and vimentin right after detachment, as in (**A**). Arrows indicate co-localized vimentin with internal integrin β1. Scale bar, 10 μm. **C** After vimentin has been re-expressed in HeLa_VIMKO cells stably by virus infection, cells were stained with anti-integrin β1 (green) and anti-vimentin antibodies (blue). The yellow box represents ROI, yellow arrows indicate vimentin-infected cells and white arrows indicate not-infected cells. Scale bar, 10 μm. Experiments shown in (**A**)-(**C**) were conducted more than twice. **D** A hypothetical diagram depicting the potential mechanism of vimentin protecting internal integrin β1 from lysosomal degradation is shown. **E** HeLa_VIMKO cells treated with or without 50 μM Bafilomycin A1 (Baf. A1) for 24 h were suspended for 0 h and stained as in (**A**). **F** The mean fluorescence of internalized integrin β1 intensities from 18 different fields was analyzed and shown as a one-dimensional scatter plot. ***, *p* < 0.0001 (pared T test)

Next, we investigated the mechanism responsible for vimentin enhancement of integrin β1 surface expression in cell suspensions. Because vimentin-dependent differential surface expression of integrin β1 was only observed in cells that had been suspended for 24 h but not immediately after suspension (Fig. 3C), we suspected integrin trafficking might be involved in the process and, thus, monitored changes in integrin β1 localization over time of suspension. When wild-type HeLa cells were stained with an anti-integrin β1 antibody immediately after cell suspension, integrin β1 was found not only in the plasma membrane but also in the cytoplasm (Fig. 5A). Suspending the cells for 12 h resulted in a decrease in internal integrin β1 staining and an increase in the cell surface staining of integrin β1 (Fig. 5A, middle). This trend was more profound in cells suspended for 24 h (Fig. 5A, bottom), confirming that the internal integrin β1 translocates to the cell surface during cell suspension. We also observed frequent co-localization of internally stained integrin β1 with vimentin (Fig. 5B, top panels, arrow). However, in HeLa_VIMKO cells, internal integrin β1 staining was not visible under any condition (Fig. 5B, bottom panels), while reintroducing vimentin into HeLa_VIMKO cells through viral infection led to the revival of internal β1 staining (Fig. 5C, yellow arrows), suggesting that vimentin may play a role in keeping internalized integrin β1. This may be due to vimentin-dependent protection of internalized integrin β1 from degradation (Fig. 5D). Indeed, when bafilomycin A1, an inhibitor of lysosomal acidification and degradation [36], was pretreated before cell detachment, integrin β1 was clearly observed inside in HeLa_VIMKO cells (Fig. 5E and F). Furthermore, in wild-type HeLa cells, treatment with bafilomycin A1 led to the co-localization of internal integrin β1 with lysosomes (Additional file 1: Supplementary Fig. 2, arrows). Conversely, in the absence of bafilomycin A1, this co-localization was not observed, suggesting the rapid lysosomal degradation of internalized integrin β1. Overall, these data suggest that vimentin protects internal integrin β1 from lysosomal degradation and maintains integrin β1 pools that can move to the cell surface during cell suspension, resulting in enhanced integrin β1 surface expression.

### Phosphorylation of Ser38 residue on vimentin enhances integrin β1 surface expression

**Fig. 6:**
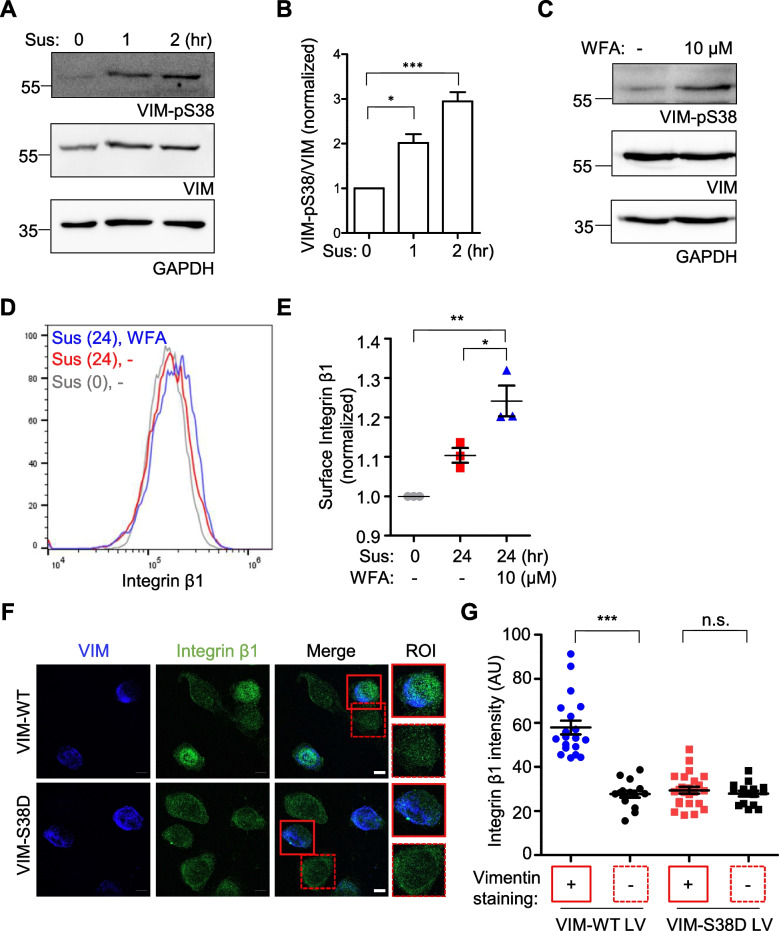
Regulation of integrin β1 localization through vimentin Ser38 phosphorylation. **A** HeLa cells suspended for 0, 1, and 2 h were analyzed by western blot using an anti-phospho-Ser38 specific vimentin antibody (VIM-pS38) as well as an anti-vimentin antibody (VIM) and an anti-GAPDH antibody (GAPDH) as a control. **B** The ratios of phosphorylated vimentin to total vimentin expression at each time point were normalized against those from cells suspended for 0 h and presented as a bar graph. ∗ , *p* < 0.05, ***, *p* < 0.0001 (one-way ANOVA followed by Tukey multiple-comparison test, *n* = 3). **C** HeLa cells suspended in the presence of 10 μM withaferin A (WFA) for 2 h were analyzed as in (**A**). **D** HeLa cells suspended for 0 or 24 h in the absence or presence of 10 μM WFA were analyzed for surface integrin β1 by flow cytometry. **E** The mean fluorescence intensities of integrin β1 staining in each condition were normalized against the 0-h control and presented as a one-dimensional scatter plot. ∗ , *p* < 0.05, ∗  ∗ , *p* < 0.001 (one-way ANOVA followed by Tukey multiple-comparison test, *n* = 3). **F** HeLa_VIMKO cells were infected by virus bearing either vimentin wild-type (upper panels, VIM-WT) or vimentin (Ser38Asp) mutant cDNA (bottom panels, VIM-S38D). After infection, cells were detached and stained with anti-vimentin (blue) and anti-integrin β1 (green) antibodies. The boxes represent regions of interest (ROI) that have been digitally magnified on the right. The boxes with dotted line and with solid line indicate not-infected cells and vimentin-infected cells, respectively. **G** The mean fluorescence of internal integrin β1 intensities in cells infected ( +) or uninfected cells (-) with vimentin wild type or S38D mutant were shown as a one-dimensional scatter plot. **, *p* < 0.001, n.s., not significant (pared T test)

We subsequently investigated the trafficking initiation of internal integrin β1 to the plasma membrane upon cell detachment. Based on our previous observations that the vimentin-integrin β1 interaction is mediated by the Ser38 residue on the head domain of vimentin and that modifications such as phosphorylation of the residue disrupt the interaction [19], we hypothesized that phosphorylation of the Ser38 residue could be the signal for releasing the internal integrin β1 from vimentin, enabling its translocation to the plasma membrane. This hypothesis was in agreement with the increased vimentin Ser38 phosphorylation observed after cell suspension (Fig. 6A and B). In addition, artificial induction of vimentin Ser38 phosphorylation by withaferin A (Fig. 6C), a natural product known to induce the phosphorylation [37], also enhanced integrin β1 surface expression (Fig. 6D and E). Furthermore, in HeLa_VIMKO cells transduced with either wild-type vimentin or vimentin mutant bearing Asp instead of Ser in the 38th residue, we observed internal integrin β1 exclusively in cells expressing wild-type vimentin (Fig. 6F, solid line in the top panel), whereas non-infected cells (Fig. 6F, dotted line in the top panel) and vimentin mutant-infected cells (Fig. 6F, solid line in the bottom panel) did not show such internal staining. This observation was further quantified by measuring the fluorescence intensities of integrin β1 staining in infected and non-infected cells for each infection condition (Fig. 6G).

### Inhibition of vimentin Ser38 phosphorylation reduces metastasis

**Fig. 7:**
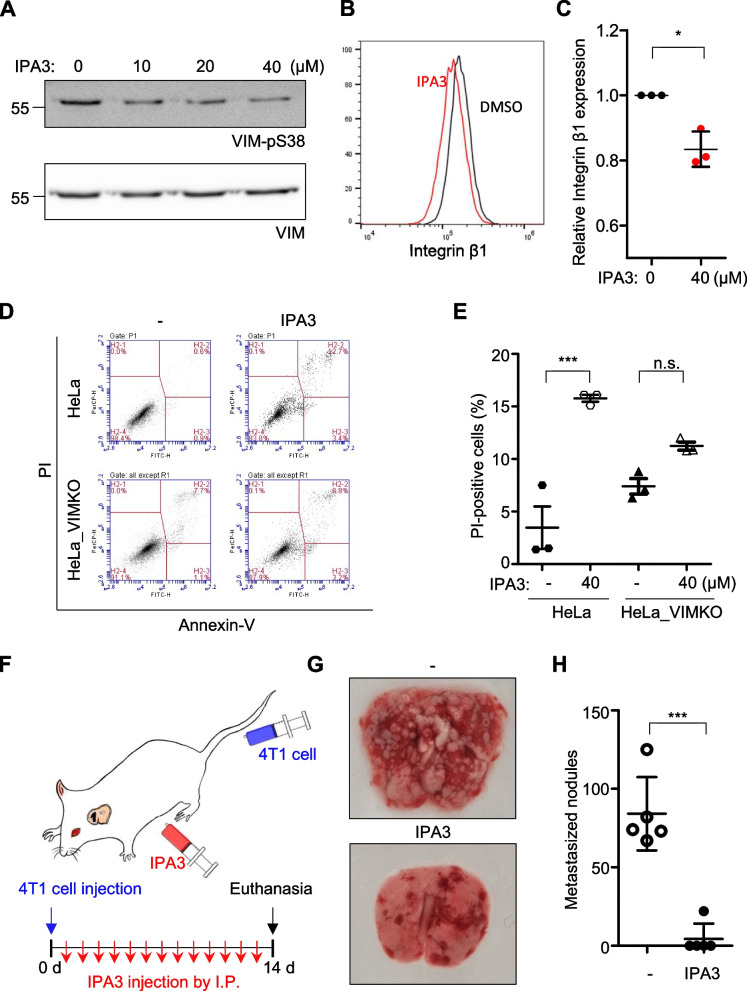
Effects of inhibiting vimentin S38 phosphorylation on metastasis. **A** HeLa cells were suspended for 2 h in the presence of varying concentrations of IPA3 as indicated, and Ser38 phosphorylation of vimentin was measured as in Fig. 6A. **B** HeLa cells suspended for 2 h in the presence or absence of 40 μM IPA3 were analyzed for surface integrin β1 by using flow cytometry. **C** The mean fluorescence intensities of integrin β1 staining in each condition were normalized against the untreated control and presented as a one-dimensional scatter plot. *, *p* < 0.05 (paired T test, *n* = 3). **D** HeLa and HeLa_VIMKO cells suspended for 24 h in the presence or absence of 40 μM IPA3 were analyzed by flow cytometry as in Fig. 1A. **E** Percentages of PI-positive cells in (**D**) are shown. ***, *p* < 0.0001, n.s.: not significant (one-way ANOVA followed by Tukey multiple-comparison test, *n* = 3). **F** Schematic of an in vivo experiment using BLAB/c mice. 1 × 10.^6^ cells of 4T1 were injected into the tail vein, and 5 mg/kg IPA3 or DMSO were treated through intraperitoneal injection every day for 14 days. **G** Representative pictures of lungs from mice treated with control vehicle (top) or IPA3 (bottom) are shown. **H** The number of metastatic nodules in the lungs in each group was counted and presented as a graph. ***, *p* < 0.0001 (unpaired T test, *n* = 5 for each group)

The above results cumulatively suggest that vimentin prevents internal integrin β1 from lysosomal degradation, and its Ser38 residue is phosphorylated upon cell detachment, allowing the trafficking of integrin β1 to the cell surface, which, in turn, promotes cell survival signaling protecting suspended cells from anoikis. If this hypothesis is proven to be correct, blocking vimentin Ser38 phosphorylation would impair the survival of circulating tumors and could potentially serve as a cancer metastasis prevention strategy. Therefore, we used the selective inhibitor IPA3 [38, 39] to inhibit PAK1, a kinase responsible for vimentin Ser38 phosphorylation [20, 40]. When HeLa cells were treated with IPA3, the suspension-induced vimentin Ser38 phosphorylation and the surface expression of integrin β1 were markedly inhibited (Fig. 7A, B, and C) at 2 h of cell suspension. As a result, IPA3 treatment during 24 h cell suspension significantly increased the anoikis cell death (Fig. 7D and E).

Finally, to validate our findings in an animal model, we utilized the 4T1 syngeneic mouse model in which BALB/c mice and 4T1 breast cancer cell line originating from the same strain are used [41]. The survival benefit of wild type 4T1 cells compared to vimentin knockout 4T1 (4T1_VIMKO) made by CRISPR-Cas9 (Additional file 1: Supplementary Fig. 3A and B) were similar to those of HeLa (Additional file 1: Supplementary Fig. 3C and 3D). Mice were injected through their tail veins with 1.0 × 10^6^ cells, a higher number than typically used in experiments, either from 4T1_VIMKO or its parental wild-type 4T1, and their abilities to form lung metastatic nodules were monitored two weeks later. While metastatic nodules were extensively formed on the lung surface of mice injected with wild-type 4T1 cells, such nodules were barely observed in 4T1_VIMKO-injected mice (Additional file 1: Supplementary Fig. 3E), confirming the role of vimentin in successful metastasis. Importantly, within this model, daily intraperitoneal injection of IPA3 (Fig. 7F) almost completely blocked lung metastasis of 4T1 cells (Fig. 7G, H), highlighting the potential benefit of blocking Ser38 phosphorylation for treating cancer metastasis.

## Discussion

In this study, we demonstrate for the first time the pro-metastatic role of vimentin in increasing the survival of suspended cells. Based on the results of the present study, the following detailed mechanism underlying the anoikis resistance caused by vimentin contribution is proposed (Additional file 1: Supplementary Fig. 4). First, vimentin protects the internal integrin β1 pool from lysosome-dependent degradation (Fig. 5 E and F) in a Ser38 residue-dependent manner (step 1). When cells detach from rigid ECMs and become suspended, the Ser38 residue is phosphorylated (Fig. 6A) (step 2). This Ser38 phosphorylation disrupts the integrin-vimentin interaction, allowing the trafficking of internal integrin β1 to the cell surface (Fig. 6E) (step 3). Increased integrin β1 on the cell surface enhances cell–cell clustering (Fig. 3A) (step 4), which induces an integrin-dependent survival signal (Fig. 4E) despite the absence of rigid extracellular contact, facilitating better survival than cells without such clustering (step 5).

Endocytosis and the recycling of integrins are essential for cell migration. Integrins located in pre-existing adhesion sites are detached from, the cell rear, and supplied to new adhesion sites, for example, at the front, enabling a continuous cycle of detachment and adhesion during cell migration [42, 43]. Alternatively, internalized integrins can be transported to lysosomes for degradation, as has been reported for fibronectin-bound integrin α5β1 [44, 45]. The large amount of internal integrin β1 detected upon bafilomycin A1 treatment in our study (Fig. 5E) suggests that a considerable amount of the integrin β1 pool was degraded in suspended cells. Because the vimentin-integrin β1 interaction occurs at the N-terminal head domain [18] that is not involved in polymerization, vimentin filaments would expose many highly concentrated nonstructural head domains with high affinity for internalized integrin β1. Thus, it is reasonable to assume that vimentin filaments underneath the plasma membrane can capture internalized integrins before they reach lysosomes, thereby preventing their lysosomal degradation, and store them until needed. In this regard, we propose that vimentin can be considered as an “integrin buffer” regulating the cellular level of β1 integrins, without which internalized integrin get destroyed quickly.

The directional transport of integrin β1 in highly polarized migratory cells has been shown to be facilitated by the *trans*-Golgi network oriented towards the leading edge, which captures the internalized β1 through retrograde trafficking and resecrets in a polarized manner [46]. Similarly, it seems that the vimentin buffering function proposed in our study can be used as an alternative route for such directional supply of integrin β1. A pioneering study suggested that vimentin Ser38 phosphorylation in response to migration cues such as Rac1 activation leads to the formation of lamellipodia at the front sites of migrating cells [47]. As Ser38 is the key residue for the vimentin-integrin β1 interaction [19], phosphorylation of the residue at the leading edge can release vimentin-interacting integrins, allowing their translocation to the plasma membrane to form lamellipodia. Thus, timely Ser38 phosphorylation at the appropriate location would function as an exit mechanism for integrin pools, allowing them to translocate from the buffer to the cell surface. Indeed, we showed that withaferin A, the well-known inducer of vimentin Ser38 phosphorylation (Fig. 6C, [37]), increases the β1 surface expression (Fig. 6E). This exit mechanism is highly relevant to an insightful study that showed that oligomeric vimentin is associated with integrin containing endocytic vesicles until vimentin N-terminal serine residues are phosphorylated by Protein kinase C epsilon [48].

Our findings indicate that metastatic cancers exploit the buffering function of vimentin to increase surface integrin β1 levels, thereby enhancing cell survival in suspension. Inhibition of PAK1, the primary downstream target of Rac1 [49] responsible for vimentin Ser38 phosphorylation [40, 50] greatly reduced metastasis (Fig. 7), highlighting a promising potential for developing drugs targeting this function for cancer treatment. However, the exact mechanism by which the cell suspension induces the phosphorylation of vimentin Ser38 residues (Fig. 6A) and the involvement of other vimentin kinases, such as protein kinase C [48, 51] and protein kinase A [50], in this process remains unclear, and further investigations are necessary for its elucidation, which will be addressed in future studies.

## Conclusions

In conclusion, this study revealed that vimentin can trap and protect internalized integrin β1 from degradation and can enhance integrin-mediated cell clustering formation, leading to better cell survival by releasing the integrin pool upon cell detachment. Thus, this study not only provides evidence for a novel mechanism of action of vimentin’s pro-metastatic function but also highlights the potential of using the inhibition of vimentin Ser38 phosphorylation, the switch for releasing integrin pools, as an efficient therapeutic target to block metastasis.

## Methods

### Cell culture

HeLa cells, HeLa_VIMKO, 4T1 cells, and 4T1_VIMKO were maintained in growth media containing Dulbecco’s modified Eagle’s medium (HyClone, SH30243.01) supplemented with 10% (v/v) fetal bovine serum (Gibco, 16,000–044) and 1% penicillin–streptomycin (HyClone, SV30010) at 37 °C in a CO_2_ incubator. For generating vimentin knockout cells, lentiCRISPR v2 (Addgene, #52,961) was used with 5’-CAACGACAAAGCCCGCGTCGAGG-3’ and 5’-CCATGTCTACCAGG-3’ target sequences for HeLa and 4T1 cells, respectively, as previously described [52].

### Antibodies

Anti-phospho-AKT (cat. no. 4051S) anti-AKT (cat. no. 9272), anti-cleaved PARP (cat. no. 9541), anti-fibronectin (cat. no. 26836), and anti-vimentin (cat. no. 5741S) were purchased from Cell Signaling Technology. The anti-integrin β1 antibodies (sc-365679 for western blot and sc-13590 for flow cytometry and cytochemistry) and the anti-GAPDH antibodies (sc-32233) were purchased from Santa Cruz Biotechnology. The anti-LAMP-1 antibody (cat. No. NB120-19294) was purchased from Novus Biologicals.

### Single-cell RNA sequencing data analysis

Single-cell RNA sequencing data for pancreatic tumor cells were downloaded from the Gene Expression Omnibus (GSE51372). Circulating tumor cells were selected using published meta-data [31]. PCA was performed using the Seurat package [53]. Reactome analysis was performed using the analyse_sc_clusters function of the ReactomeGSA package [54].

### Fluorescence microscopy

Cells were treated with 50 nM bafilomycin A1 (Sigma, B1793) for 24 h or transfected with wild-type or mutant (Ser38Asp) vimentin cDNA constructs using polyJet (Signagen, SL100688) accordingly. The cells were detached by trypsinization and suspended in growth media containing poly-HEMA (Sigma, P3932-10G)-coated surfaces for 0–24 h. The suspended cells were washed with phosphate-buffered saline (PBS) and then attached to the silane-coated glass slide by spinning down using a Cytospin 4 Centrifuge (Thermo Scientific, TH-CYTO4) at 250 × g. The attached cells were then fixed, permeabilized, and stained using anti-integrin β1, anti-fibronectin, and/or anti-vimentin antibodies. Alexa Fluor 488 goat anti-mouse IgG (Invitrogen, A11001), Alexa Fluor 350 goat anti-rabbit IgG (Invitrogen, A11046), Alexa Fluor 405 goat anti-rabbit IgG (Invitrogen, A31556), rhodamine-conjugated Goat Anti-Rabbit IgG (Jackson ImmunoResearch, 111–025-003), and 4′,6-diamidino-2-phenylindole (DAPI; VECTOR LAB, H-1200) were used accordingly. Images of the stained cells were captured using a confocal microscope (ZEISS, LSM800) and processed using Zen Blue (Zeiss) or ImageJ software (National Institute of Health).

### Flow cytometry

1.5 × 10^5^ cells suspended on a poly-HEMA-coated surface for 0 to 24 h in the presence or absence of 20 ~ 40 μM RGDS peptides (RnD system, 3498–10), 10 ~ 40 μM IPA3 (Sigma, I2285), or 10 μM withaferin A (APExBio, B7199). Cells were washed and stained with PI (Sigma, P4170) and FITC-conjugated annexin-V (AAT-Bioquest, 22,839). Occasionally, the cells were stained with an anti-integrin β1 antibody, followed by Alexa Fluor 488 goat anti-mouse IgG (Invitrogen, A11001). Stained cells were analyzed by flow cytometry (BD Biosciences, FACS Accuri C6). Alternatively, suspended cells were analyzed by western blotting.

### Adhesion and spreading assays

Cells suspended for 24 h were then incubated on a 20 μg/ml fibronectin (Sigma, F2006)- or 0.01% poly-L-lysine (Sigma, P4707)-coated surface for 40 min. The degree of cell adhesion was measured using the CCK8 assay (Dojindo, 3000 T) according to the manufacturer's instructions. Alternatively, the adhered cells were stained with phalloidin (Invitrogen, R415), and images were captured using a fluorescence microscope (Nikon, Eclipse Ti-E) to measure their spread area.

### Lung metastasis

7-week-old female BALB/c mice were purchased from Raon Bio (Seoul, Korea). 10^6^ 4T1 cells or 4T1_VIMKO cells suspended in 100 µl PBS were injected into the mouse tail veins. IPA3 was intraperitoneally injected daily at a dose of 5 mg/kg for two weeks. Mice were finally sacrificed with carbon dioxide, and isolated lungs were frozen at − 80 °C before picture capture. All animal studies were approved by the Institutional Animal Care and Use Committee of Korea University (KUIACUC-2022–0097).

### Statistical Analysis

Statistical analysis was performed on data from more than three independent experiments using GraphPad Prism5 (GraphPad Software, USA). Data are presented as mean ± SEM according to statistical tests indicated in the figure legends. Other statistical parameters, including n numbers and *p* values, are indicated in the figure legends.

### Supplementary Information

Additional file 1: Supplementary Figs. 1–4. Supplementary Fig. 1. Generation of vimentin knockout cells. Supplementary Fig. 2. Integrin β1 targeted to lysosomes in detached cells. Supplementary Fig. 3. Survival benefit of vimentin in 4T1 Cells. Supplementary Fig. 4. Hypothetical model of the integrin buffering function of vimentin.

Additional file 2: Supplementary images of the original, uncropped blots.

## Data Availability

All data generated or analyzed during this study are included in this published article and its supplementary information files, or are available from the corresponding author on reasonable request. The single-cell RNA sequencing data for pancreatic tumor cells were downloaded from the Gene Expression Omnibus (GSE51372) [31].

## Abbreviations

EMT: Epithelial-to-mesenchymal transition
ECM: Extracellular matrix
HeLa_VIMKO: Vimentin knockout HeLa
poly-HEMA: Poly-2-hydroxyethyl methacrylate
PI: Propidium iodide
PARP: Poly(ADP-ribose) polymerase
CTC: Circulating tumor cells
RGDS: Arg-Gly-Asp-Ser
4T1_VIMKO: Vimentin knockout 4T1
PBS: Phosphate-buffered saline
DAPI: 4’,6-Diamidino-2-phenylindole
