# Supplementary material for: Vimentin-mediated buffering of internal integrin β1 pool increases survival of cells from anoikis
Source: BMC Biol. 2024 Jun 24;22:139. doi: 10.1186/s12915-024-01942-w (PMC11197373; doi:10.1186/s12915-024-01942-w)
Supplement: Supplementary file 1 — Additional file 1: Supplementary Figs. 1–4. Supplementary Fig. 1. Generation of vimentin knockout cells. Supplementary Fig. 2. Integrin β1 targeted to lysosomes in detached cells. Supplementary Fig. 3. Survival benefit of vimentin in 4T1 Cells. Supplementary Fig. 4. Hypothetical model of the integrin buffering function of vimentin. [file 12915_2024_1942_MOESM1_ESM.pdf]

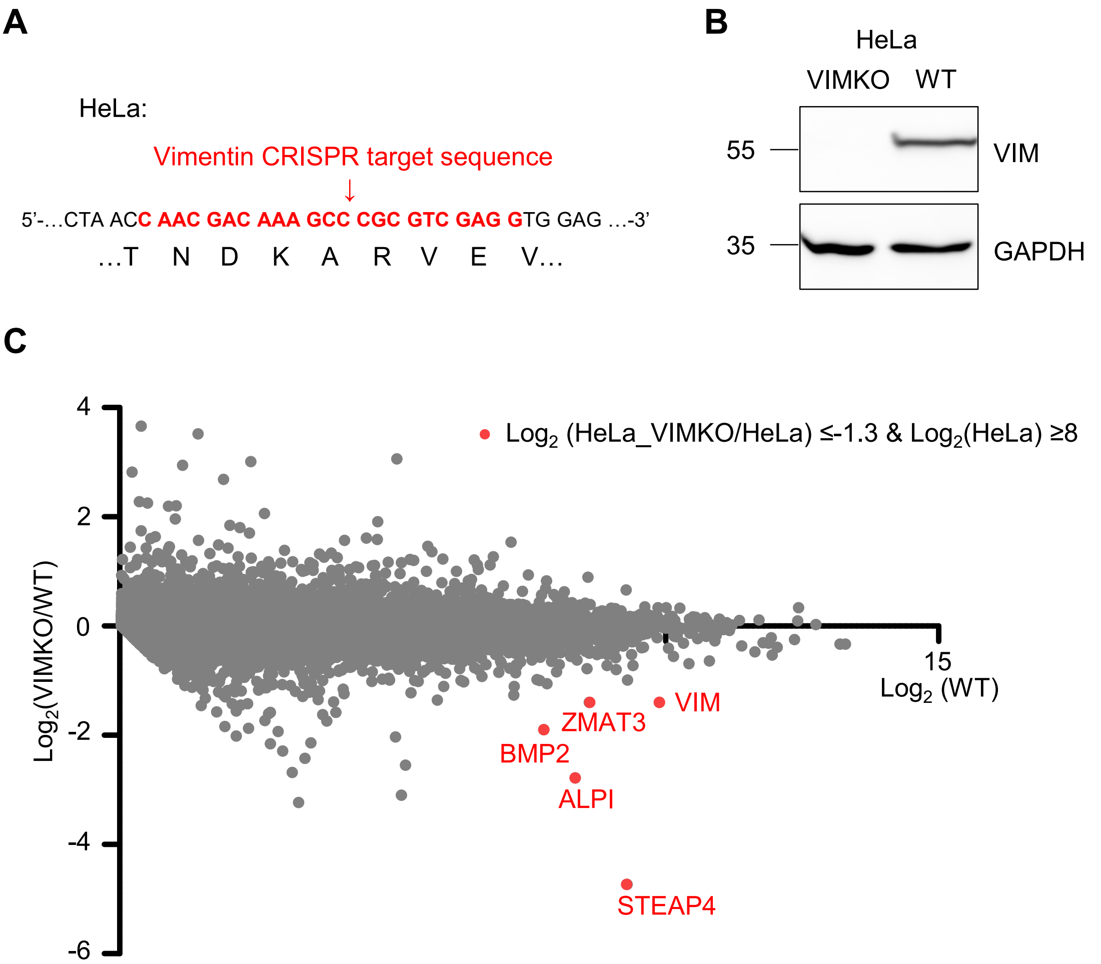

D

Gene symbol	Log_2 (HeLa)	Log_2(HeLa_VIMKO/HeLa)	Similarity (CAACGACAAAGCCCGCGTCGAGG)
VIM	9.883523196	-1.39901631	100%
STEAP4	9.291627559	-4.732430912	No significant similarity found.
ZMAT3	8.608455983	-1.396413814	
ALPI	8.348008754	-2.775822243	

Supplementary Figure 1. Generation of vimentin knock-out cells. (A) The part of the human vimentin cDNA sequence and the CRISPR target region for generating HeLa_VIMKO cells are shown. (B) The absence of vimentin in HeLa_VIMKO cells was confirmed by western blot using anti-vimentin antibody (VIM) and anti-GAPDH antibody (GAPDH) as a control. (C) Differentially Expressed Genes (DEG) analysis using mRNA sequencing from HeLa and HeLa_VIMKO cells and results were displayed as MA plot. *x*-axis is log_2(WT) which means log_2(mean of normalized count of HeLa cells), and *y*-axis is log_2(VIMKO/WT) which means log_2(fold change) and ford change implies mean of normalized count of HeLa-VIMKO cells to that of HeLa cells. (D) The table shows the red dots in (C) and indicates no significant similarity to the vimentin CRISPR target sequence.

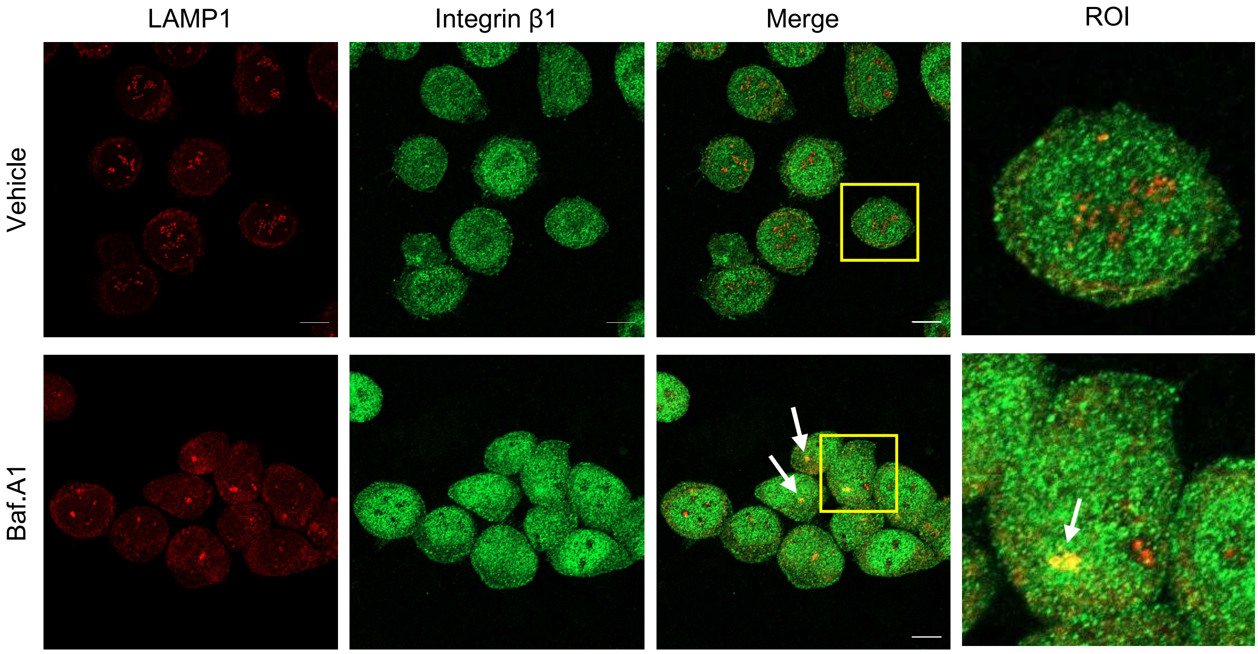

Supplementary Figure 2. Integrin β1 targeted to lysosomes in detached cells. After HeLa cells were treated with vehicle (upper panel) or 50 μM Bafilomycin A1 (Baf. A1, lower panel) for 24 hours, cells were detached and stained with anti-integrin β1 (green) and anti-LAMP1 (red) antibodies. Yellow boxes show region of interest (ROI) and arrows indicate co-localized with integrin β1 and LAMP-1.

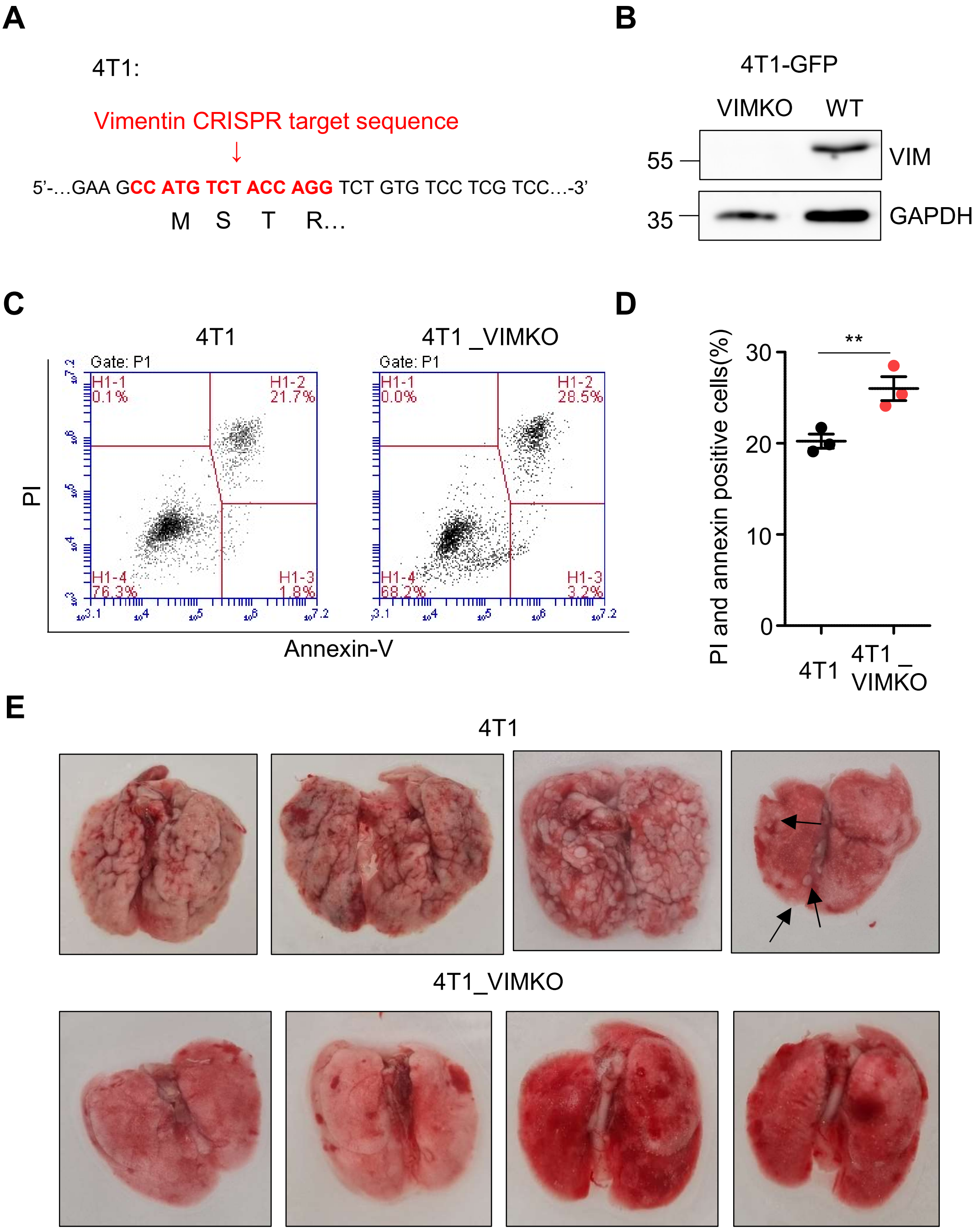

Supplementary Figure 3. Survival benefit of vimentin in 4T1 Cells. (A) The part of the mouse vimentin cDNA sequence and the CRISPR target region for generating 4T1_VIMKO cells are shown. (B) The absence of vimentin in 4T1_VIMKO cells was confirmed by western blot using anti-vimentin (VIM) and anti-GAPDH (GAPDH) antibodies. (C) 4T1 and 4T1_VIMKO cells suspended for 24 hours were analyzed as in Fig. 1A. (D) Percentages of PI-positive cells in (C) are shown as a graph. **, $p < 0.001$, (paired T-test, $n = 3$). (E) BALB/c mice were tail-vein injected with 10^6 4T1 cells or vimentin knock-out 4T1 (4T1_VIMKO) cells (4 mice per each condition). Lungs from all mice were visualized after 2 weeks. Arrows indicate metastatic nodules. We note that almost all the surfaces of the lungs shown in the top three panels were covered with metastatic nodules.

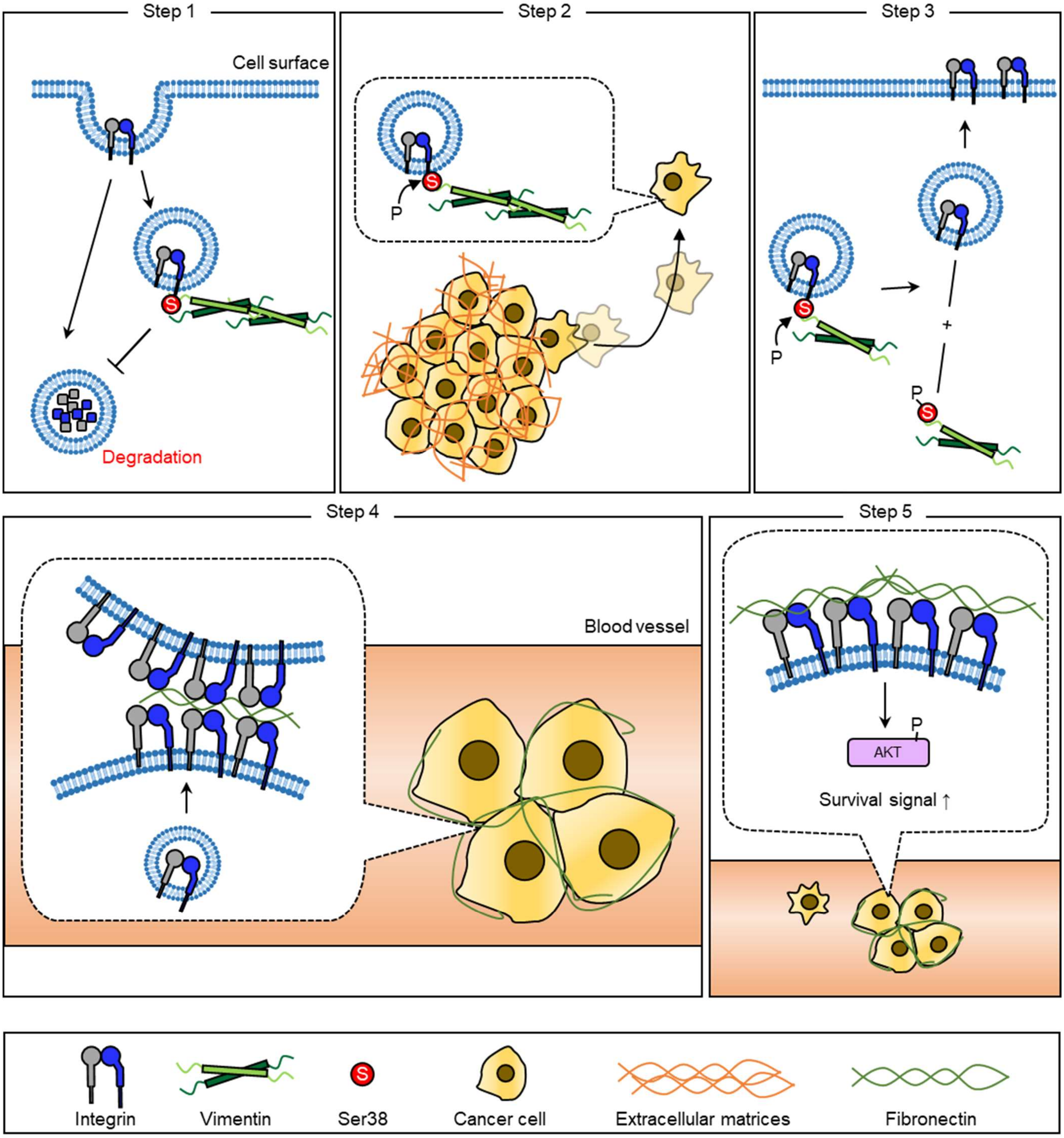

Supplementary Figure 4. Hypothetical model of the integrin buffering function of vimentin. Internalized β1 integrins are degraded or captured by vimentin (Step 1). Upon cell detachment at the initial stage of metastasis, vimentin is phosphorylated at Ser38 (step 2). Vimentin Ser38 phosphorylation inhibits the vimentin-integrin β1 interaction, causing the movement of internalized β1 integrins to the cell surface (step 3). Increased integrin β1 on the cell surface enhances cell-cell clustering (step 4). In clustered cells, an integrin-dependent survival signal increases, resulting in resistance to anoikis (Step 5).
