# Supplementary material for: Vimentin-mediated buffering of internal integrin β1 pool increases survival of cells from anoikis
Source: BMC Biol. 2024 Jun 24;22:139. doi: 10.1186/s12915-024-01942-w (PMC11197373; doi:10.1186/s12915-024-01942-w)

Additional file 2: Uncropped blot Figure 1C

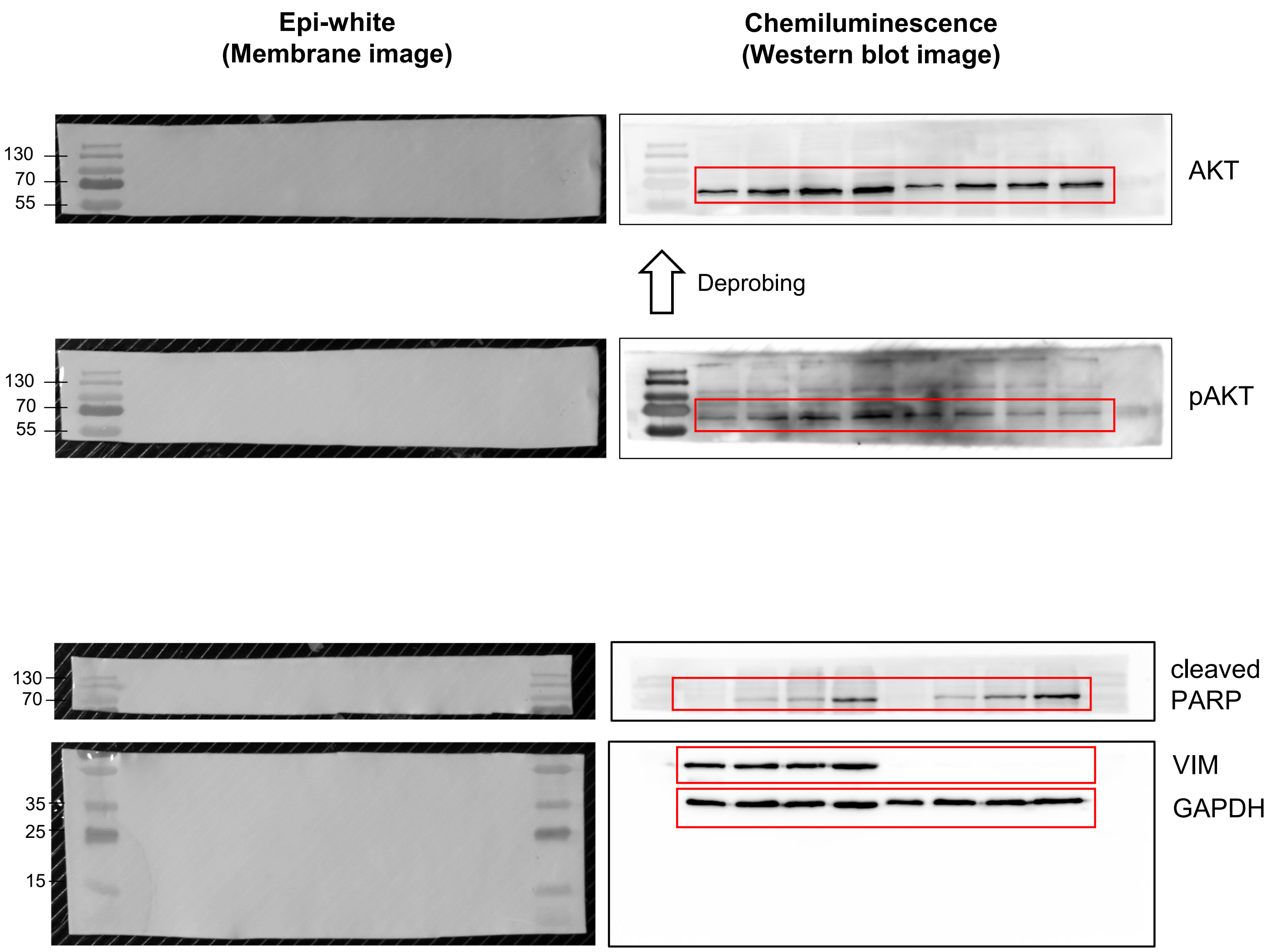

Additional file 2: Uncropped blot Figure 4E

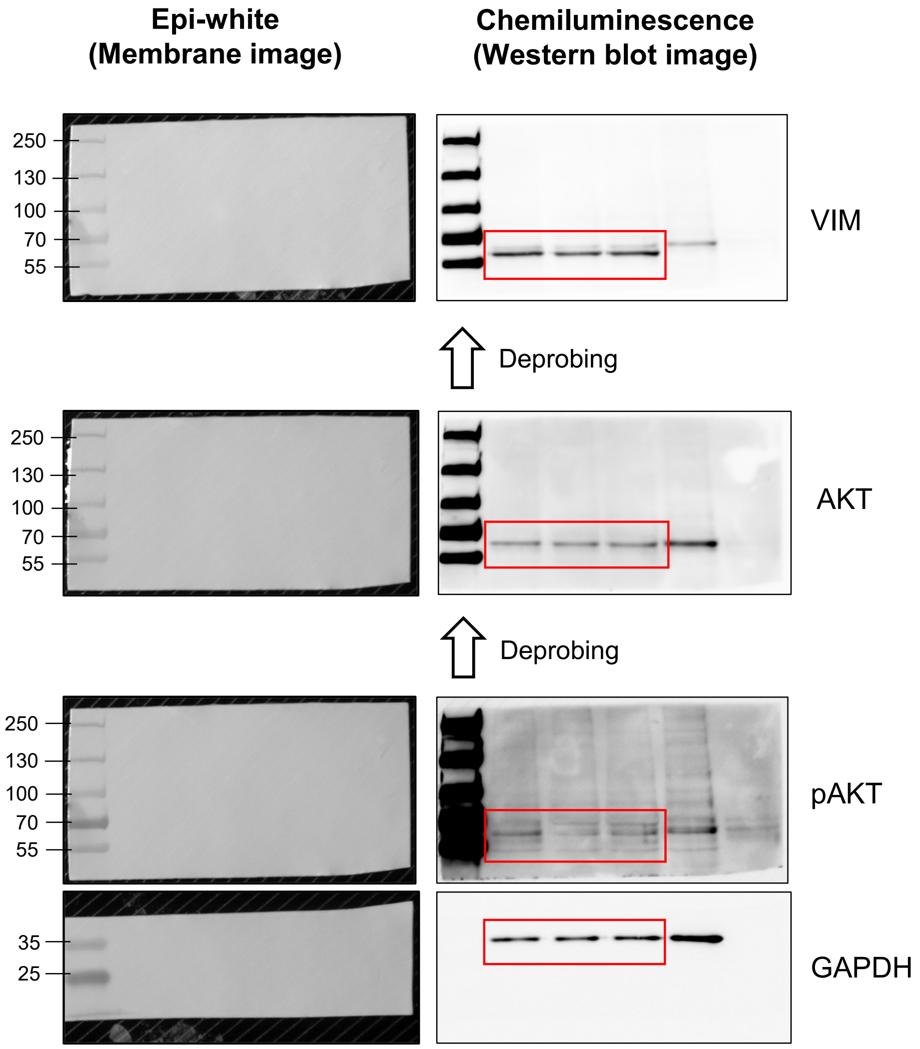

Additional file 2: Uncropped blot Figure 6A

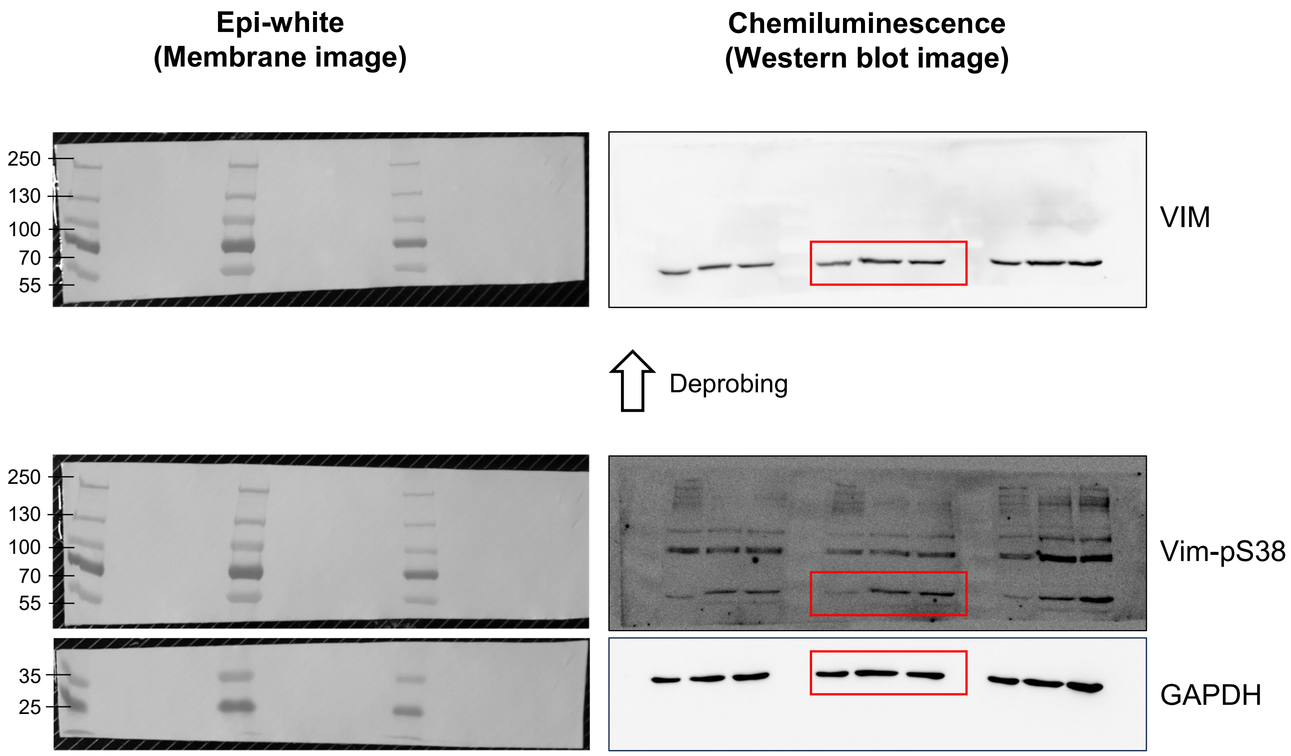

Additional file 2: Uncropped blot Figure 6C

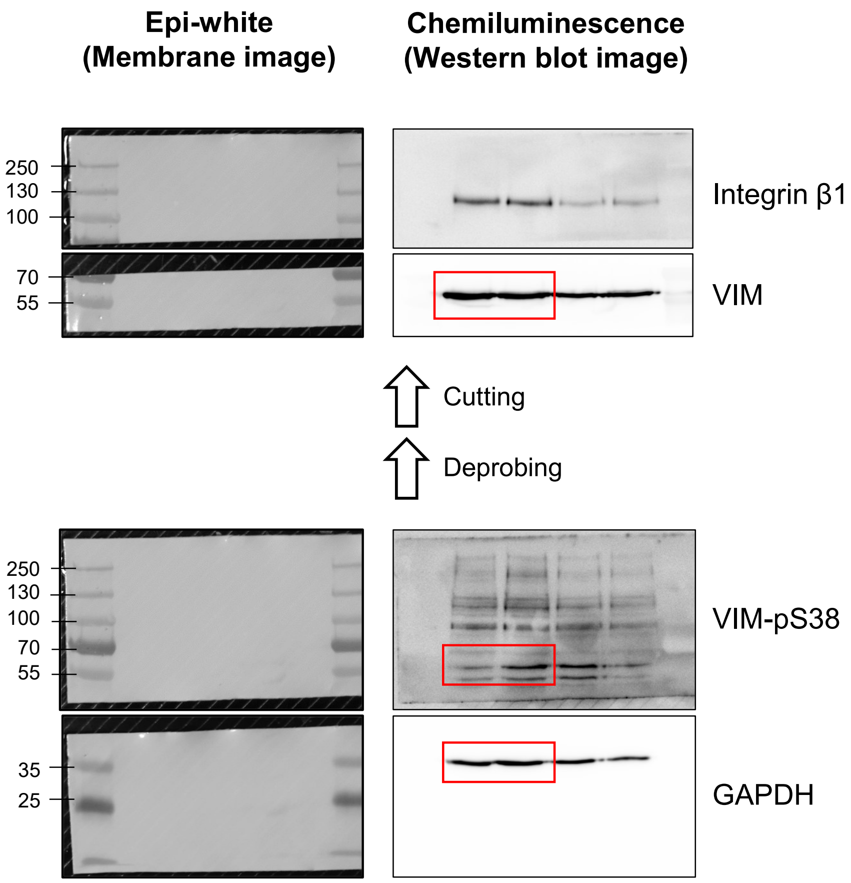

Additional file 2: Uncropped blot Figure 7A

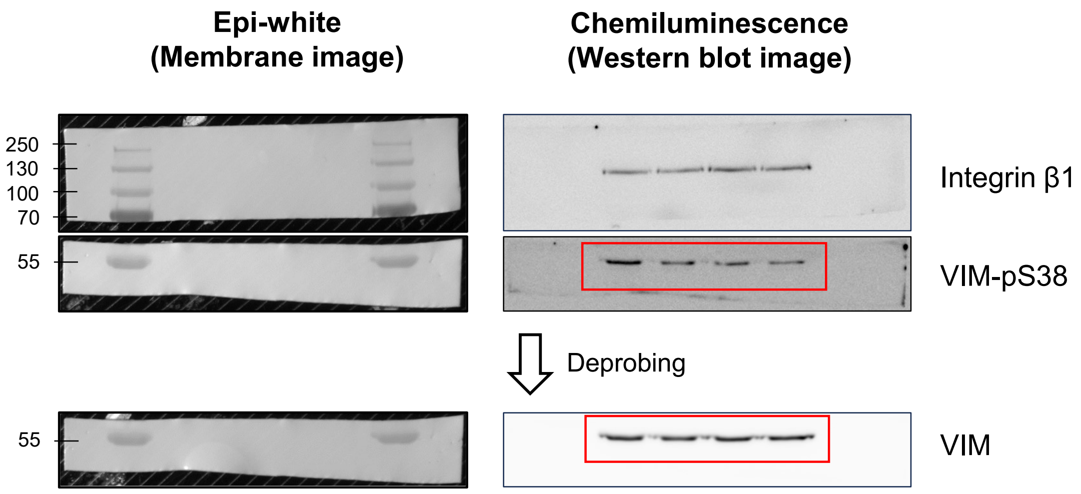

Additional file 2: Uncropped blot Supplementary Figure 1B

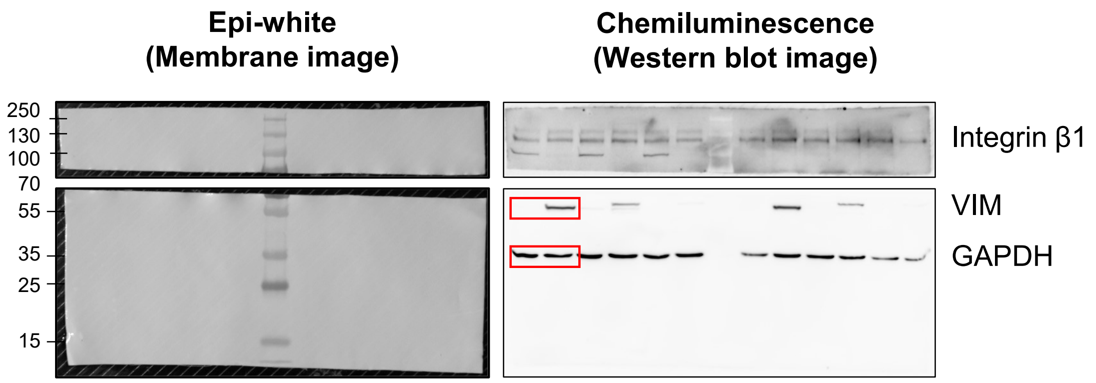

Additional file 2: Uncropped blot Supplementary Figure 3B

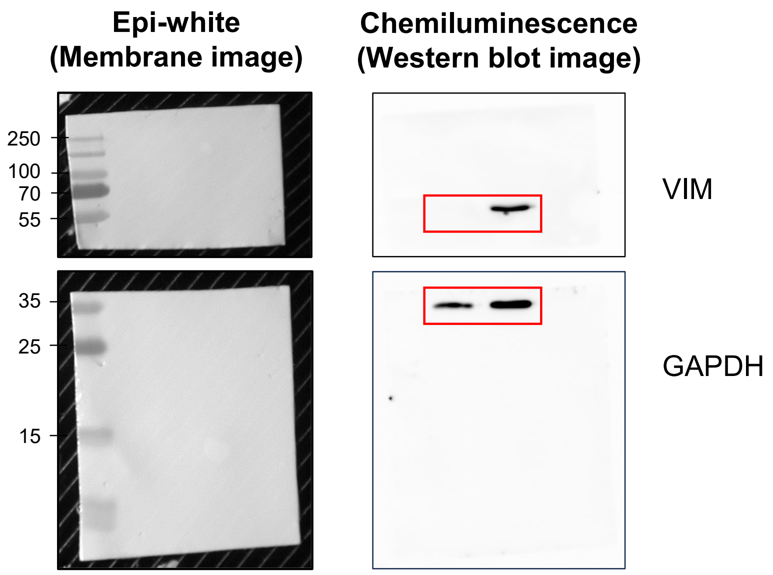

Supplement: Supplementary file 2 — Additional file 2: Supplementary images of the original, uncropped blots. [file 12915_2024_1942_MOESM2_ESM.pdf]
